# Non-lethal heat shock unlocks *SOD* gene family diversity for enhanced bacterial resistance in *Procambarus clarkii*

**DOI:** 10.3389/fimmu.2026.1713713

**Published:** 2026-01-21

**Authors:** Xin Zhang, Xiuhong Cai, Shirui Yue, Zhangxuan Chen, Lei Cheng, Shunchang Wang

**Affiliations:** 1 School of Biological Engineering, Huainan Normal University, Huainan, Anhui, China; 2 Anhui Huaihe River Basin Center for Aquatic Animal Epidemic Disease Protection and Control, Huainan, Anhui, China

**Keywords:** crust aceanimmunity, endoplasmic reticulum stress, non-lethal heat shock, *Procambarus clarkii*, SOD gene family

## Abstract

**Background:**

Oxidative stress and endoplasmic reticulum stress (ERS) are critical for crustaceans’ stress responses. Genome-wide identification of superoxide dismutase (SOD) genes in *Procambarus clarkia* is essential for understanding its stress adaptation and aquaculture disease control.

**Methods:**

Five PcSOD genes were identified, with analyses of their structure, motif, chromosomal distribution and phylogeny. Their tissue-specific expression, expression under *Vibrio parahaemolyticus* challenge, correlation with ERS-related genes, and changes in T-AOC/SOD activity were detected, along with the effect of heat shock pretreatment.

**Results:**

PcSOD genes showed structural diversity and tissue specificity, with time-dependent expression under bacterial challenge. Heat shock pretreatment regulated their expression timing and intensity. Significant correlations between PcSOD and ERS genes were observed in hemocytes under NLHS + V. *parahaemolyticus* treatment, supported by NLHS-induced Hsp70.

**Conclusion:**

These findings suggest a potential coordinated SOD-ERS response in *P. clarkia*, providing insights for aquaculture disease control strategies.

## 1 Introduction

Superoxide dismutase (SOD) is a key enzyme in the biological antioxidant defense system. It maintains cellular redox homeostasis by catalyzing the dismutation of superoxide anion (O_2_^2^) into oxygen (O_2_) and hydrogen peroxide (H_2_O_2_) (1, 2). Based on metal cofactors, the family includes CuZnSOD, MnSOD, FeSOD, and NiSOD—with CuZnSOD and MnSOD being functionally crucial in eukaryotes (3–5). Under pathogen or environmental stress, SOD precisely regulates reactive oxygen species (ROS) accumulation to balance host defense and self-protection (4, 6–8). For example, *ecCuZnSOD* expression is significantly upregulated in *Litopenaeus vannamei* infected with *Vibrio alginolyticus*, with enzyme activity positively correlated with disease resistance (9). SOD3 in *Acipenser baikalensis* is also rapidly induced under *Aeromonas hydrophila* stimulation, highlighting its critical role in mucosal immunity (10). Additionally, SOD family expression is linked to environmental adaptability—*Cyprinus carpio* upregulates sod genes synergistically with gpx to mitigate cadmium-induced oxidative damage (11).

The endoplasmic reticulum (ER), as the central hub for protein synthesis and folding, is vital for maintaining cellular homeostasis in aquatic animals (12). Thermal stress from temperature fluctuations disrupts ER function in ectothermic species, triggering the unfolded protein response (UPR). UPR impairs protein synthesis, induces misfolded protein accumulation and oxidative stress, and disrupts mitochondrial function, immunity, and energy metabolism—ultimately reducing feed efficiency and increasing disease susceptibility. SOD is involved in modulating ER stress (ERS) (12), with excess ROS under stress altering SOD activity or expression to influence ERS levels. For instance, xiaoaiping exposure reduced SOD activity and increased ERS gene expression in zebrafish embryos (13). Nitrite exposure induced ERS and antioxidant imbalance in *L. vannamei* (14), while Ulva prolifera exposure reduced hepatic SOD activity and induced ERS in *Paralichthys olivaceus* (15). Under carbonate alkalinity stress, SOD activity in shrimp gills showed a transient rise followed by a decline, while ERS genes remained elevated (16). In crucian carp, beta-cypermethrin exposure caused dynamic SOD changes linked to ERS pathway activation (17). These findings highlight the importance of SOD–ERS balance in stress adaptation, where imbalance may aggravate cellular damage.

As key taxa in aquatic ecosystems and important targets of aquaculture, crustaceans are of great interest in studies of the *SOD* gene family, which plays a critical role in innate immunity and environmental adaptation. Recent progress has been made in crustacean *SOD* gene cloning and functional analysis. For example, *ecCuZnSOD* expression is upregulated upon pathogenic challenge in *Portunus trituberculatus*, associated with enhanced immune protection (18) and *Eriocheir sinensis* (19), correlating with enhanced immune protection and pathogen clearance. These findings confirm the conserved immune role of crustacean *SOD* genes, but comprehensive family identification, evolutionary analysis, and regulatory network characterization remain unexplored.

*Procambarus clarkii* is one of the most widely cultured crayfish species globally, known for its rapid growth and strong environmental adaptability (20). However, its production and quality are compromised by pathogenic infections and environmental stresses such as temperature fluctuations (21). Previous work cloned two *P. clarkii SOD* genes (*ecCuZnSOD* and *mtMnSOD*) from hemocytes, showing high expression in hepatopancreas, gills, and hemocytes (22). Their expression and enzymatic activities increased significantly under pathogenic stimulation, confirming their immune role (22). Nevertheless, this study only identified two *SOD* members and lacked comprehensive family-wide analysis. In contrast, research on *Callinectes sapidus* has emphasized that complete identification of the *SOD* gene family is essential to understand functional redundancy and divergence (23). Additionally, the interaction between abiotic stresses and pathogen infection—common in aquaculture—remains uninvestigated. The lack of genome-wide analysis limits understanding of the family’s evolutionary patterns, gene structures, and regulatory elements, hindering insights into its functional network (22).

Non-lethal heat shock (NLHS) can enhance tolerance to subsequent stress by inducing heat shock proteins (HSPs) and increasing antioxidant enzyme activity. In *L. vannamei*, NLHS upregulated *HSP70* and immune-related transcripts but did not improve resistance to *V. harveyi*, suggesting a complex relationship between thermal stress and pathogen defense (24). In *Artemia franciscana*, thermal acclimation regulated stress- and immunity-related genes, potentially enhancing resilience (25). These effects may result from HSPs stabilizing intracellular conditions and maintaining protein function (26, 27), plus modulation of antioxidant systems (28). In our prior study, we identified fifteen *P. clarkii Hsp70* genes via genome-wide analysis (29). We found NLHS induces their upregulation, which enhances resistance to *V. parahaemolyticus* by regulating the TLR signaling pathway—revealing Hsp70’s role in NLHS-enhanced immunity. However, whether NLHS enhances *P. clarkii* resistance to *V. parahaemolyticus* through regulating SOD family expression and ERS responses remains unknown. Genome-wide identification of the *SOD* family is essential to elucidate its roles in antioxidant defense, ERS regulation, and immune response. It enables comprehensive analysis of gene members, structures, chromosomal distribution, and evolutionary relationships, laying a foundation for understanding functional divergence (11). In common carp, for instance, genome-wide mining revealed significant expansion of the *gpx* family, with subtype-specific responses to cadmium stress, providing insights into antioxidant gene diversity (11). Transcriptome integration can further map SOD expression across tissues, developmental stages, and combined stresses, revealing regulatory networks (30). This would not only address gaps in gene identification in *P. clarkii* but also identify molecular targets underlying NLHS-enhanced immunity, supporting stress-resilient breeding and healthy aquaculture.

Based on our prior finding that NLHS induces *Hsp70* upregulation to enhance *P. clarkii*’s resistance to *V. parahaemolyticus*, we proposed this exploratory hypothesis: NLHS may modulate specific *PcSOD* expression and their interaction with the ERS pathway, forming an antioxidant-stress network to improve bacterial resistance. To validate this, we aimed to: (1) identify *P. clarkii* SOD family via genomic tools and characterize their structure, evolution, and tissue expression; (2) establish a NLHS+*V. parahaemolyticus* model to detect *SOD* expression dynamics; (3) analyze SOD-ERS interaction to clarify their role in “heat preconditioning–immune enhancement.” This work enriches crustacean SOD function knowledge, reveals *P. clarkii*’s immune mechanisms, and provides a basis for optimizing aquaculture stress/disease strategies.

## 2 Materials and methods

### 2.1 Identification and characterization of *SOD* gene family members in *P. clarkii*

The genomic assembly and corresponding annotation files of *P. clarkii* (accession number: GCA_040958095.1) were retrieved from the NCBI database (https://www.ncbi.nlm.nih.gov/). To identify candidate SOD family members, the Hidden Markov Model (HMM) profile of the Sod_Cu domain (PF00080), Sod_Fe_N domain (PF00081), Sod_Fe_C domain (PF02777) were obtained from the Pfam database (https://www.ebi.ac.uk/interpro/entry/pfam/). This HMM seed file was employed as a query using HMMER software (version 3.2; http://hmmer.org/) to screen the annotated protein-coding sequences in the *P. clarkii* genome for potential Sod domain-containing proteins. In parallel, SOD protein sequences from *E. sinensis*, *Drosophila melanogaster*, and *L. vannamei* were retrieved from the NCBI database and used to conduct BLASTP searches against the *P. clarkii* protein dataset, with an e-value cutoff of 1e−5, to ensure comprehensive identification. All candidate sequences were then cross-validated for the presence of the conserved SOD domain using the Conserved Domain Database (CDD; https://www.ncbi.nlm.nih.gov/Structure/bwrpsb/bwrpsb.cgi) and Simple Modular Architecture Research Tool (SMART) (http://smart.embl-heidelberg.de/). Sequences lacking the characteristic domain were excluded from further analysis. The final set of confirmed SOD family members was analyzed using the ExPASy ProtParam tool (http://web.expasy.org/protparam/) to determine their primary physicochemical properties, including amino acid length, molecular weight (MW), and theoretical isoelectric point (pI).

### 2.2 Structural features of *SOD* genes and proteins in *P. clarkii*

Gene structure information, including exon–intron organization of the identified *P. clarkii SOD* genes, was extracted from the genome annotation file in GFF3 format using TBtools software (31). To explore the conserved structural features at the protein level, motif analysis was performed using the MEME suite (https://meme-suite.org/meme/), with the maximum number of motifs set to 10. The distribution of conserved motifs and exon–intron structures were both visualized using TBtools. For secondary structure prediction, the full-length amino acid sequences of SOD proteins were analyzed using the SOPMA online tool. Tertiary structures were modeled using the SWISS-MODEL server, and only predicted models with a sequence identity greater than 30% to their respective templates were retained for downstream analysis.

### 2.3 Chromosomal mapping and phylogenetic analysis of *P. clarkii SOD* genes

The genome annotation file of *P. clarkii* was used to determine the chromosomal positions of SOD family members, along with the corresponding chromosome lengths. This information was extracted using the “Gene Location Visualize” function within the GTF/GFF module of TBtools software. Based on these data, a chromosomal distribution map of *PcSOD* genes was constructed. For evolutionary analysis, a phylogenetic tree was generated using the Neighbor-Joining (NJ) method implemented in MEGA 6.0 software (32). The analysis was performed with 1,000 bootstrap replicates to assess branch reliability, while other parameters were maintained at their default settings.

### 2.4 Experimental animals and sample collection

*P. clarkii* individuals (body weight: 15–20 g) were obtained from a commercial aquaculture farm located in Huainan, Anhui Province. A total of 300 crayfish were used in this study. Prior to experimentation, the crayfish were acclimated under standard aquaculture conditions, including routine feeding and water quality management. After a 7-day stabilization period to establish uniform physiological baselines, 260 healthy crayfish with stable overall conditions were selected for subsequent experiments (the remaining 40 individuals were excluded as backups due to minor health inconsistencies).

The experimental design consisted of two sequential phases:

Heat Shock Phase (HSS):

a) NT group (Non-Treated Control): Individuals were maintained in water at 26 °C for 6 h without any thermal treatment.
b) HT group (NLHS-Treated): Individuals were subjected to heat stress by exposure to a 32 °C water bath for 2 h, followed by a 4 h recovery at 26 °C.

Pathogen Challenge Phase (PIS):

a) HTC group: Individuals from the HT group were intramuscularly injected with 30 μL of sterile normal saline (NS).
b) HTV group: Individuals from the HT group were injected with 30 μL of *V. parahaemolyticus* suspension (1.0 × 10^8^ CFU/mL).
c) NTC group: Individuals from the NT group were injected with 30 μL of NS.
d) NTV group: Individuals from the NT group received 30 μL of *V. parahaemolyticus* (1.0 × 10^8^ CFU/mL).

At 3 h, 12 h, 24 h, and 48 h post-treatment in PIS phases, hemolymph samples were collected from six individuals per group (n = 6 per time point per group). Hemolymph was centrifuged at 2000 × g for 10 minutes at 4 °C. The supernatant was discarded, and the hemocyte pellets were immediately flash-frozen in liquid nitrogen and stored for subsequent RNA extraction. Detailed information regarding the experimental groups has been elaborated in our previous study (29).

### 2.5 Tissue-specific expression profiling and expression analysis of *SOD* genes in response to bacterial infection Following NLHS

The expression profiles of five *SOD* genes across seven tissues of *P. clarkii*—hemocytes (He), gills (Gi), hepatopancreas (Hp), eyestalks (E), intestines (I), stomachs (St), and muscles (Mu)—will be characterized. Total RNA was extracted from hemocytes using TRIzol reagent (Invitrogen, USA), and RNA quality and concentration were assessed via NanoDrop 2000 spectrophotometry (Thermo Scientific, USA) and agarose gel electrophoresis. For each sample, 1 µg of total RNA was reverse-transcribed into cDNA using M-MLV reverse transcriptase (Promega, Madison, WI, USA).

Quantitative real-time PCR (qPCR) was conducted to assess gene expression levels in hemocytes subjected to various treatments. Gene-specific primers (listed in **Supplementary Table S1**) were designed to amplify target fragments of 200–300 bp. The *P. clarkii* glyceraldehyde-3-phosphate dehydrogenase (*Gapdh*; Accession No. AB094145) gene was used as the internal reference, as its stable expression under the experimental conditions was validated. PCR reactions were performed on a Roche LightCycler 96 thermal cycler using 10× SYBR Green Master Mix (Yisheng, Shanghai, China), 9 µL of diluted cDNA template, and 0.5 µL of each primer (10 μM). Thermal cycling conditions included an initial denaturation at 95 °C for 1 minute, followed by 40 amplification cycles of 95 °C for 15 seconds and 60 °C for 1 minute. A melting curve analysis was performed post-amplification to confirm the specificity of the PCR products. Relative expression levels were calculated using the 2^–ΔΔCT method for data normalization (33).

All reactions were carried out in triplicate. For statistical analysis: 1) For tissue-specific expression profiling, differences in gene expression across tissues were evaluated using one-way analysis of variance (ANOVA) followed by Tukey’s HSD *post-hoc* test with IBM SPSS Statistics 20; 2) For pathogen-induced expression analysis (group comparisons under infection conditions), differences were assessed using t-test via the same software. A *p*-value < 0.05 was considered statistically significant. To visualize expression profiles, a heatmap was generated using R software (v4.5.0). In addition, to further corroborate the expression patterns of *SOD* genes and their potential roles in immune regulation, previously obtained RNA-Seq data (PRJNA1107630), generated under identical experimental conditions, were re-analyzed.

### 2.6 Expression patterns of ERS-related genes in response to bacterial infection following NLHS

To investigate the involvement of ERS in the immune response of *P. clarkii* under NLHS, the transcriptional expression of five key ERS-related genes—inositol-requiring protein-1 (*IRE1*), activating transcription factor 4 (*ATF4*), activating transcription factor 6 (*ATF6*), X box-binding protein 1 (*XBP1*), and eukaryotic translation initiation factor 2 (*EIF2*)—was analyzed using qPCR. Total RNA extraction, cDNA synthesis, primer design, and qPCR conditions followed the protocols described in Section 2.5. Relative expression levels were calculated using the 2^–ΔΔCT method, with *Gapdh* as the internal control. Each reaction was performed in triplicate. Statistical analysis was conducted using IBM SPSS Statistics 20, with differences considered significant at *p* < 0.05. Gene expression patterns were visualized as heatmaps generated in R (v4.5.0).

### 2.7 Measurement of SOD and T-AOC activity

Hemolymph samples were collected from *P. clarkii* individuals across different treatment groups and centrifuged at 2000 × *g* for 10 minutes to obtain the supernatant. The activity of SOD and total antioxidant capacity (T-AOC) in the supernatant was determined using a commercial assay kit (Nanjing Jiancheng Bioengineering Institute, Nanjing, China), following the manufacturer’s instructions. Absorbance values were measured at the specified wavelength using a Varioskan LUX microplate reader (Thermo Scientific, USA). All assays were performed in triplicate for each group to ensure reproducibility and accuracy.

## 3 Results

### 3.1 Genome-wide identification and functional characterization of *P. clarkii SOD* genes

A comprehensive genome-wide analysis identified five *SOD* gene members in *P. clarkii*, each annotated based on chromosomal position and gene structure (**Table 1**). The corresponding proteins displayed notable functional diversity, with predicted amino acid lengths ranging from 205 to 313 residues and molecular weights between 21.4 and 34.0 kDa. This variation implies potential specialization in stress adaptation and protein regulatory functions. Theoretical pI, calculated to range from 5.69 to 9.1, indicate that these proteins are predominantly acidic, which may influence their subcellular distribution and protein–protein interactions, particularly under fluctuating intracellular pH conditions induced by environmental stress (**Table 1**).

**Table 1:** Basic information on *SOD* family members in *P. clarki*.

Gene_Name	Gene_ID	Chromosome	Number of amino acid	Molecular weight	Theoretical pI	Instability index	Aliphatic index	Grand average of hydropathicity
*PcSOD1*	XM_045749378.2	NC_091176.1	642	70,854.91	5.73	34.11	83.99	-0.469
*PcSOD2*	XM_069336188.1	NC_091184.1	642	70,864.95	5.67	35	84.3	-0.462
*PcSOD3*	XM_045758446.2	NC_091208.1	647	71,168.32	5.28	35.85	77.6	-0.483
*PcSOD4*	XM_069332394.1	NC_091176.1	635	69,433.29	5.49	33.81	82.33	-0.457
*PcSOD5*	XM_045748732.2	NC_091151.1	638	69,882.92	5.23	37.94	77.7	-0.443

### 3.2 Conserved motifs, CDD domains, and gene structures of *P. clarkii* SOD genes

**Figure 1:**
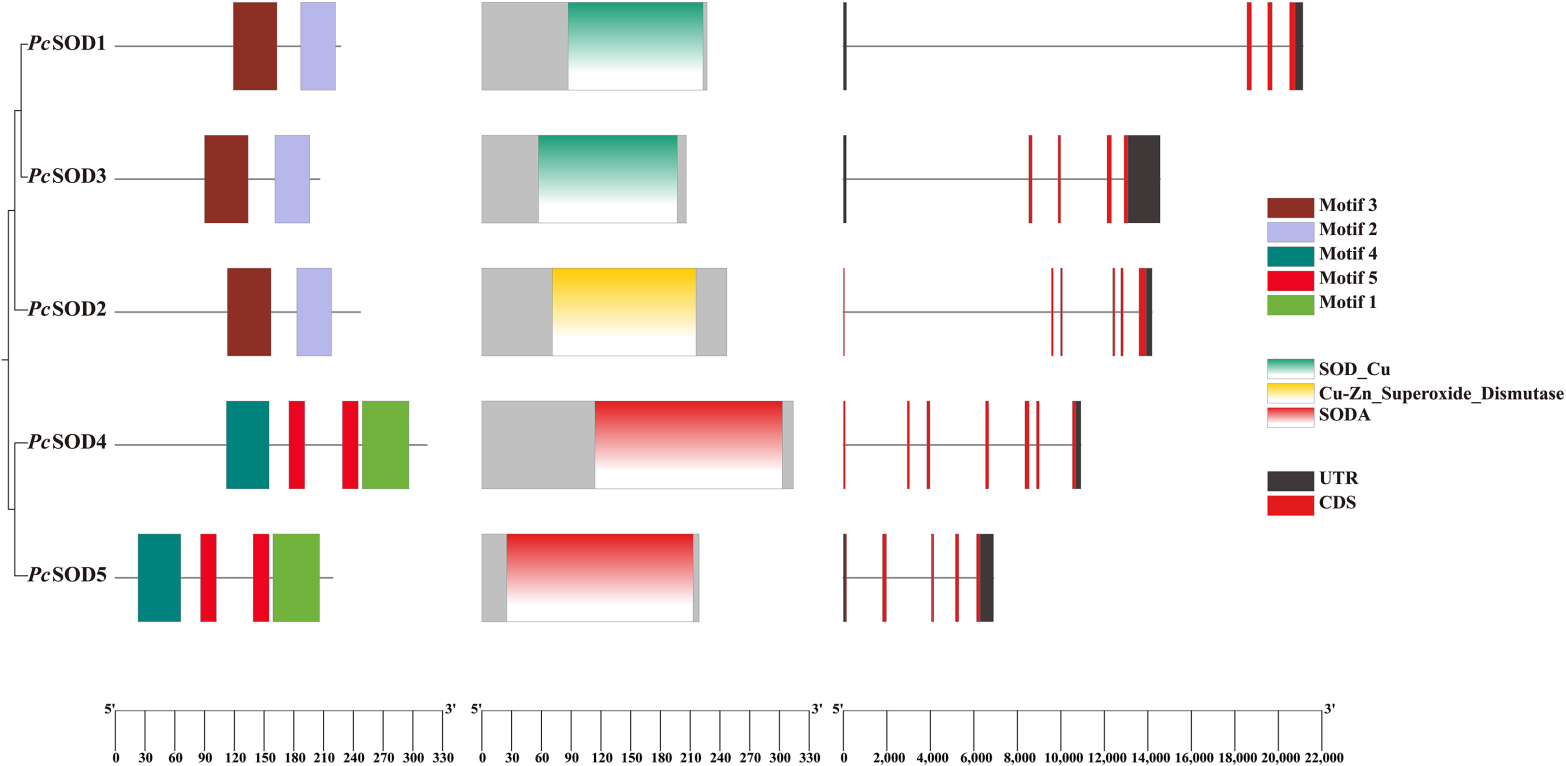
Protein domains and gene structures of the *PcSODs* family. Different motifs and domains are displayed using colorful bars. Exons and 5′ UTR/3′ UTR are displayed using black bars and red bars. Gray lines denote introns.

As shown in **Figure 1**, conserved motif analysis of *P. clarkii* SOD proteins revealed three highly conserved SOD domains present across different family members, which is essential for their enzymatic function in oxidative stress defense. These core domains reflect a shared functional mechanism, allowing these proteins to catalyze the dismutation of superoxide radicals and maintain cellular redox balance. While most motifs corresponded to this canonical SOD domain, certain variations in non-core motifs were observed among different gene members (**Figure 1**).

### 3.3 Chromosomal distribution of *P. clarkii SOD* genes

**Figure 2:**
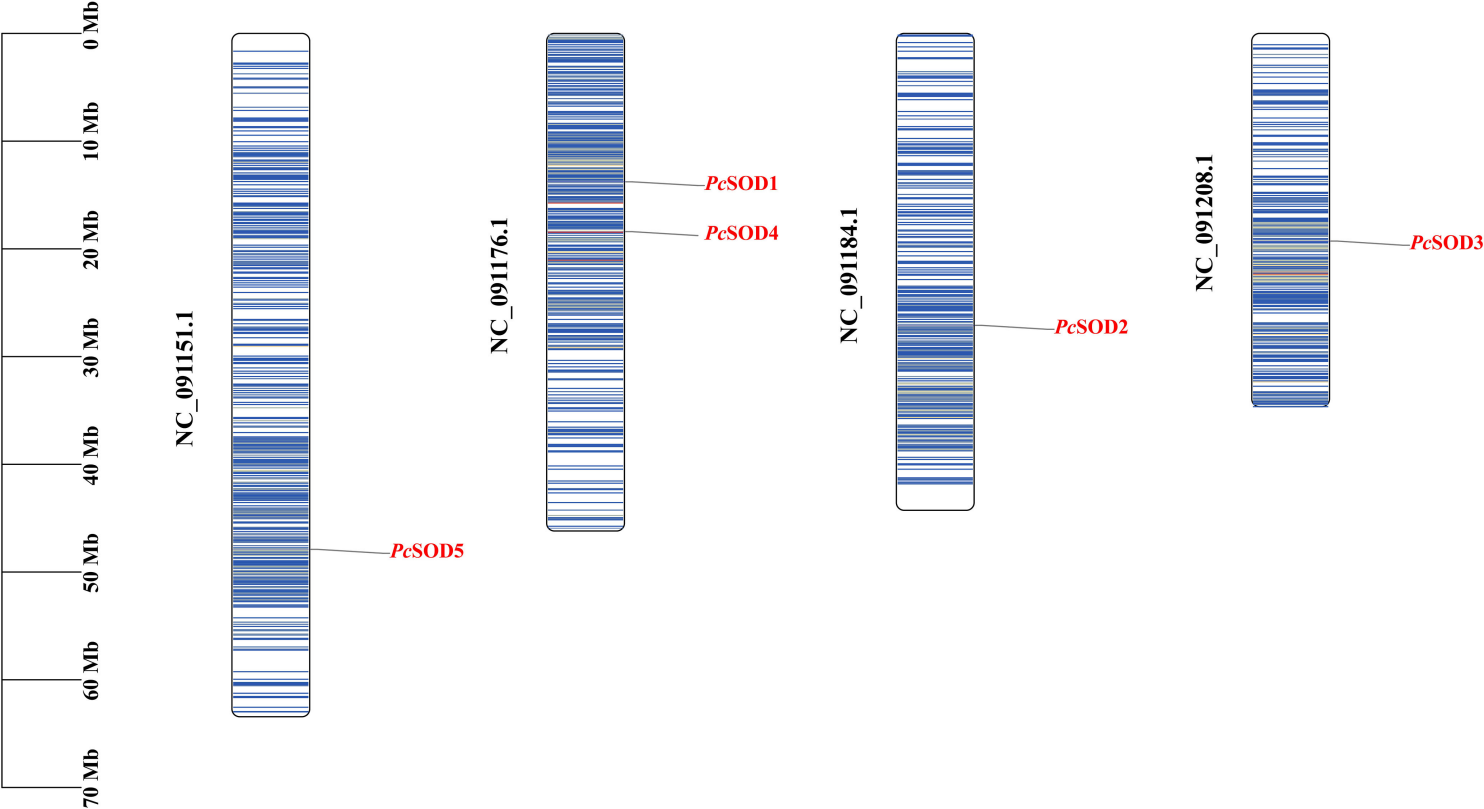
Chromosome distribution of *PcSODs*.

The chromosomal localization of *P. clarkii SOD* genes was examined, revealing that five *PcSOD* genes were unevenly distributed across four chromosomes (**Figure 2**). Most genes were dispersed at various positions without evident clustering. Among them, *PcSOD1* and *PcSOD4* were both located on chromosome NC_091176.1. The remaining three members—*PcSOD2*, *PcSOD3*, and *PcSOD5*—were individually mapped to chromosomes NC_091151.1, NC_091184.1, and NC_091208.1, respectively (**Figure 2**).

### 3.4 Structural modeling and phylogenetic analysis of *P. clarkii* SOD proteins

**Figure 3:**
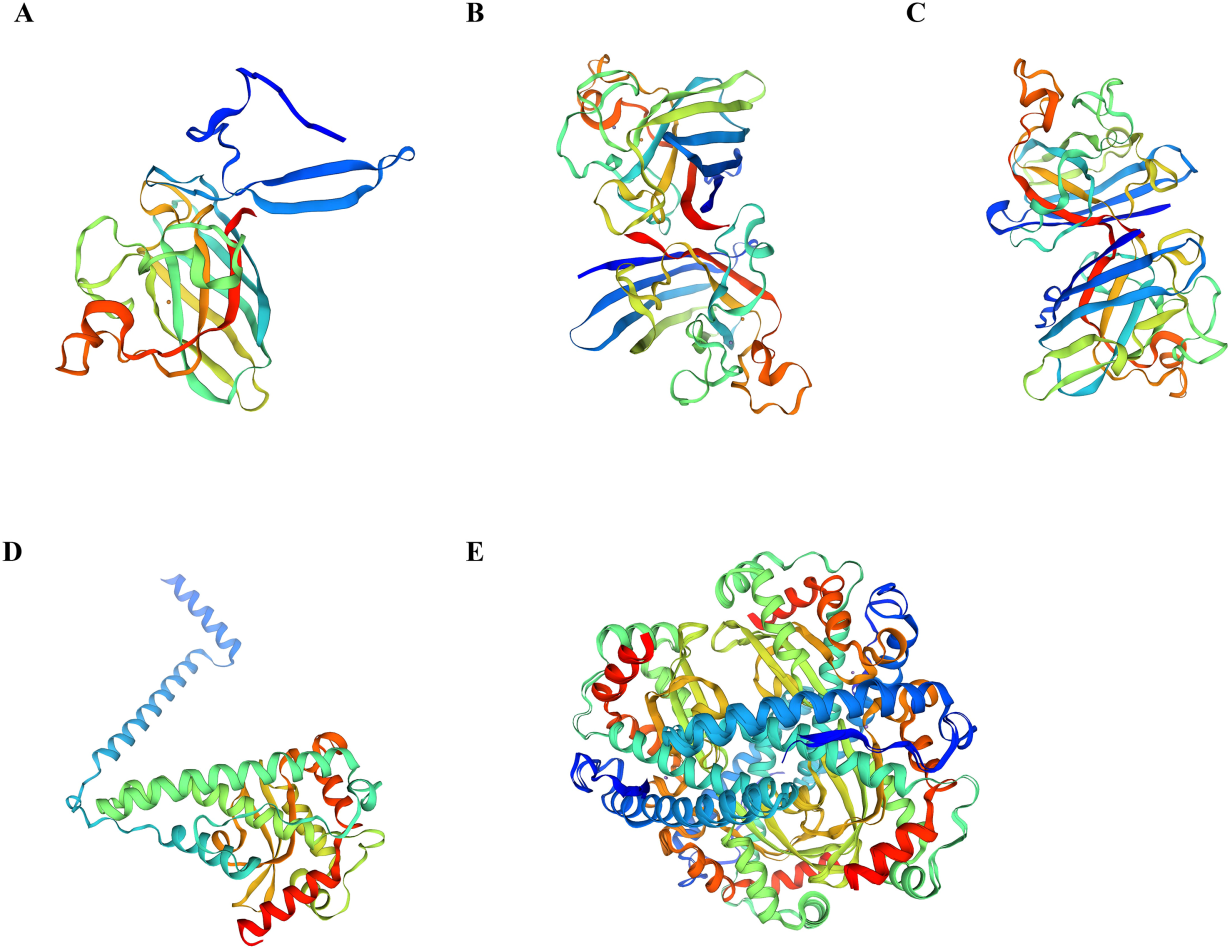
The predicted 3D structure of *Pc*SODs. **(A-E)** The 3D-structure of *Pc*SOD1, *Pc*SOD2, *Pc*SOD3, *Pc*SOD4, and *Pc*SOD5, respectively.

Homology modeling of the five identified *Pc*SOD proteins was conducted using the SWISS-MODEL server. The predicted structures revealed a conserved spatial configuration characterized primarily by α-helices and coiled regions, with several members displaying similar folding topologies. These structural similarities suggest a conserved three-dimensional framework that may reflect shared enzymatic functions or a common evolutionary origin (**Figure 3**).

**Figure 4:**
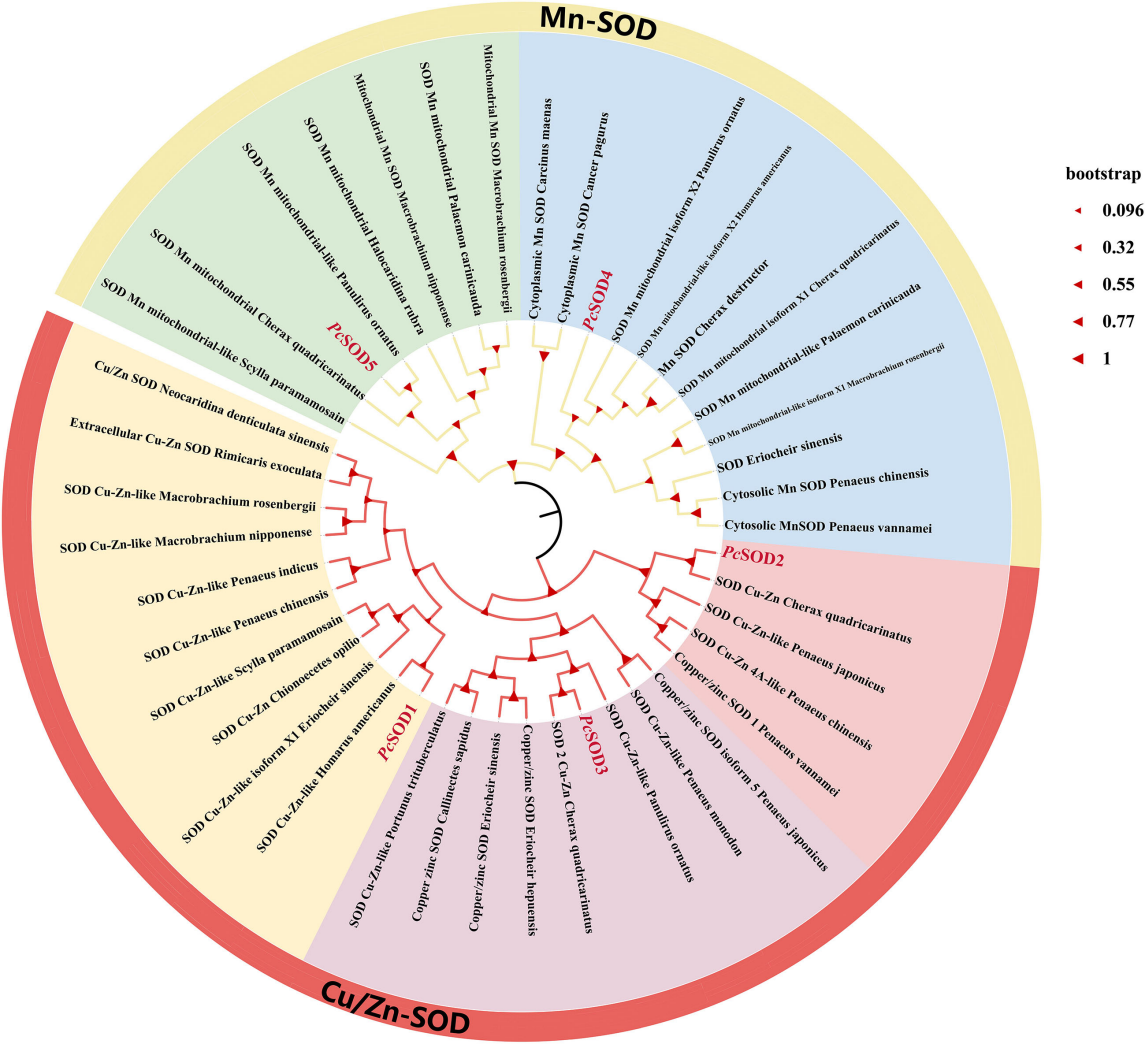
Phylogenetic analysis of *Pc*SODs from selected organisms. A phylogenetic tree was constructed by MEGA6 with the Neighbor Joining method and bootstrap of 1000 replications. Percent bootstrap values (1000 bootstrap replications) are indicated in every branch.

Phylogenetic analysis revealed that *Pc*SOD proteins clustered into two distinct subfamilies: Mn-SOD and Cu/Zn-SOD, consistent with their sequence homology and functional classification. *Pc*SOD1, *Pc*SOD2, and *Pc*SOD3 grouped with Cu/Zn-SOD proteins from various species, while *Pc*SOD4 and *Pc*SOD5 clustered closely with Mn-SOD homologs from other crustaceans. These phylogenetic patterns highlight the evolutionary divergence and taxonomic specificity within the *Pc*SOD family (**Figure 4**).

### 3.5 Expression patterns of *PcSOD* genes in different tissues

**Figure 5:**
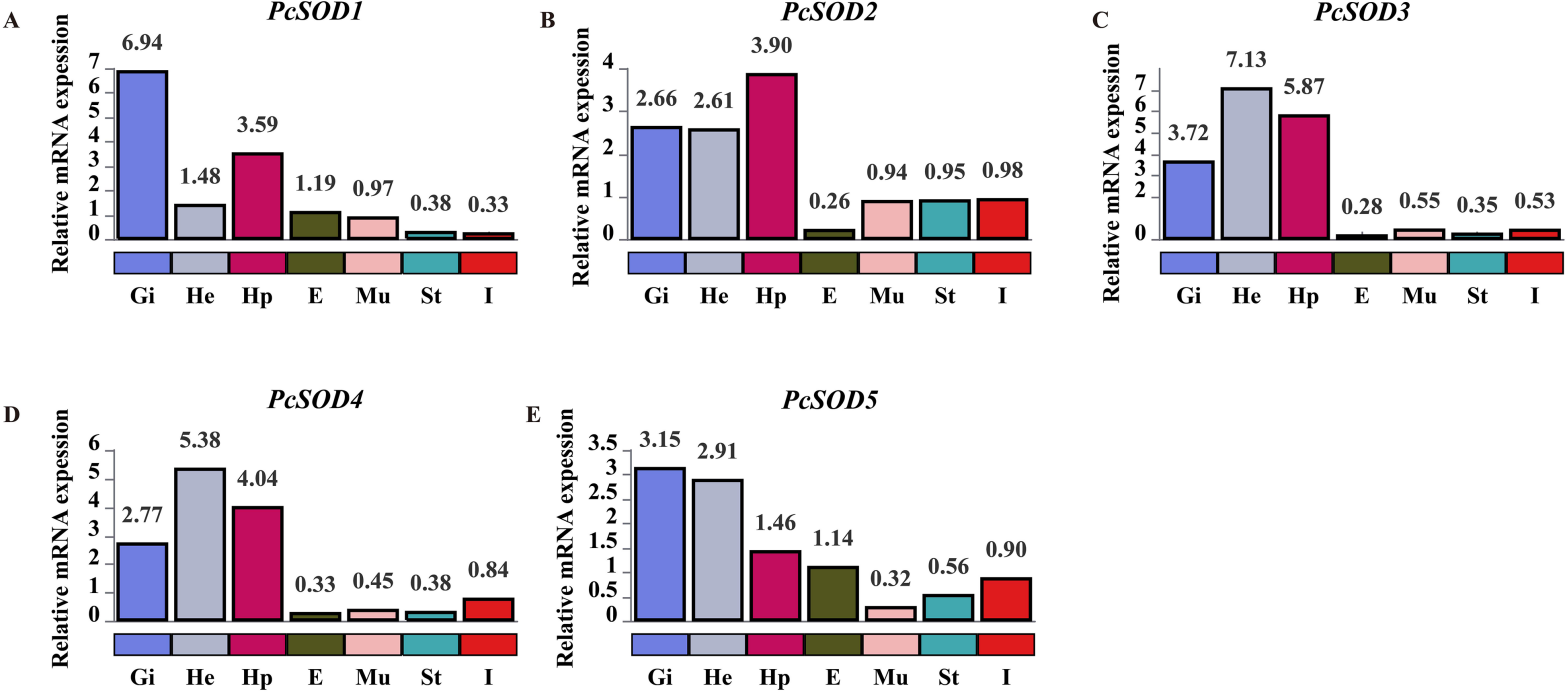
The expression profiles of *SOD* genes in hemocytes (He), gill (Gi), hepatopancreas (Hp), eyestalk (E), stomach (St), intestine (I), and muscle (Mu). **(A-E)** correspond to the expression profiles of *PcSOD1* to *PcSOD5*, respectively. Statistical analysis was performed using one-way ANOVA followed by Tukey’s HSD *post-hoc* test.

The tissue-specific expression patterns of five *SOD* genes in seven tissues of *P. clarkii* were analyzed using qPCR. As shown in **Figure 5**, distinct expression profiles were observed across different tissues. Hemocytes exhibited relatively high expression levels of multiple *SOD* genes, with *PcSOD4* showing the highest expression (5.38), followed by *PcSOD5* (2.91) and *PcSOD2* (2.61). In gill tissue, *PcSOD1* was predominantly expressed (6.94), displaying a significantly higher level compared to other genes. The hepatopancreas showed robust expression of *PcSOD3* (5.87), *PcSOD4* (4.04), and *PcSOD2* (3.90). Notably, *PcSOD4* also had detectable expression in eyestalks (0.33), albeit at a lower level than in hemocytes and hepatopancreas. Intestine-specific high expression was observed for *PcSOD5* (1.46). In contrast, *SOD* gene expression in stomach and muscle was generally low, with *PcSOD1* in stomach reaching only 0.38 and most *SOD* genes in muscle showing expression levels below 1. These results indicate that *SOD* genes in *P. clarkii* exhibit tissue-specific expression patterns, which may be associated with the distinct physiological functions and antioxidant demands of each tissue (**Figure 5**).

### 3.6 Expression analysis of *SOD* genes in response to bacterial challenge after NLHS

**Figure 6:**
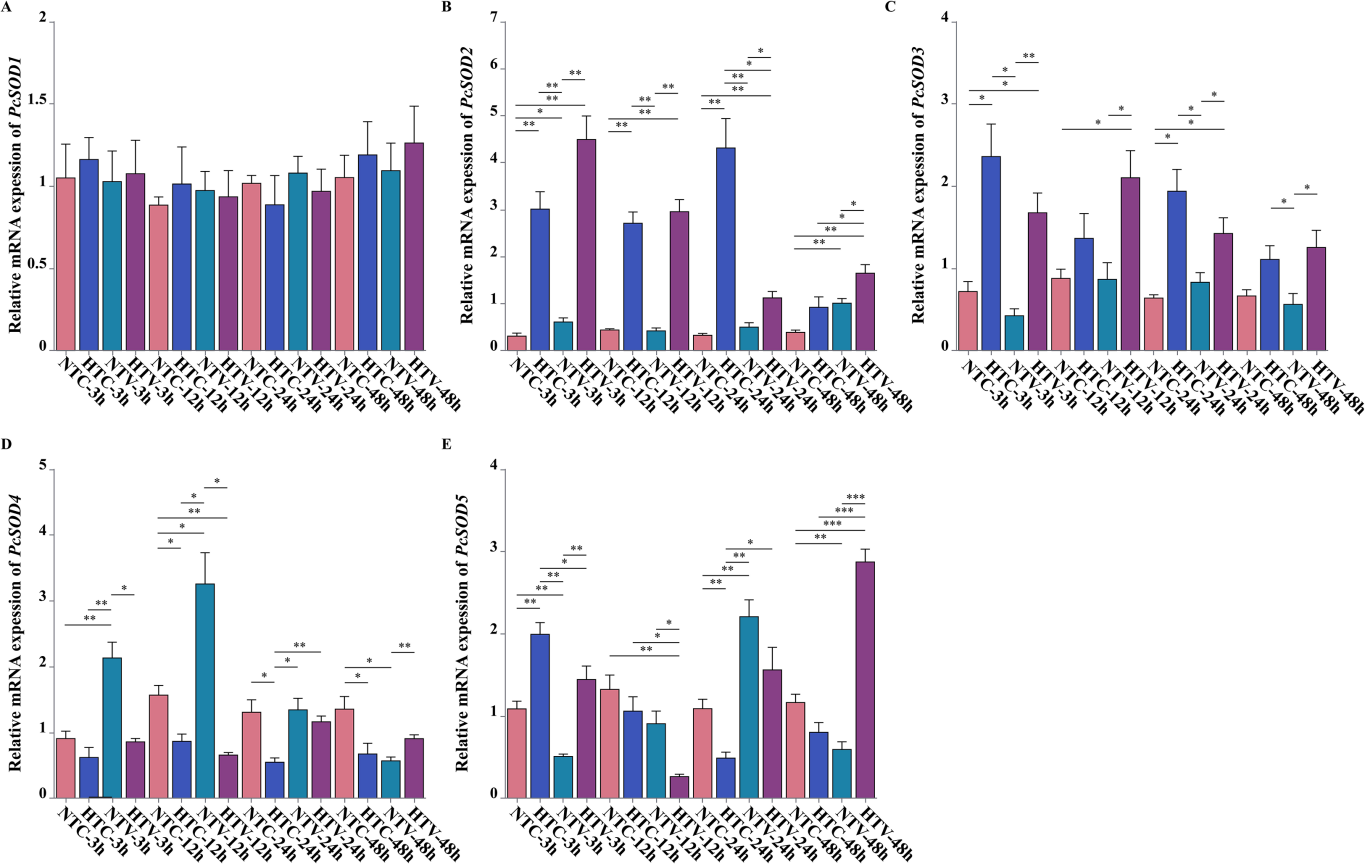
Expression dynamics of *PcSOD* genes in response to *V. parahaemolyticus* infection following NLHS. **(A–E)** qPCR analysis of *PcSOD1*, *PcSOD2*, *PcSOD3*, *PcSOD4*, and *PcSOD5* expression under different treatment groups. Expression levels were normalized to the reference gene *Gapdh*. * represents *p* < 0.05; ** represents *p* < 0.01; *** represents *p* < 0.001.

Distinct expression profiles of the five *SOD* genes were observed in hemocytes of *P. clarkii* following *V. parahaemolyticus* challenge. *PcSOD1* maintained relatively stable expression across all experimental groups, including HTC, and time points, with minimal differential regulation (**Figure 6A**). In the HTC group, the expression of *PcSOD2*, *PcSOD3*, *PcSOD4*, and *PcSOD5* remained at relatively low levels across the 3–48 h period, showing no significant fluctuations (**Figures 6B–E**). In contrast, *PcSOD2* and *PcSOD4* showed marked acute-phase responses, with significantly elevated expression at 3 h and 12 h post-challenge in the pathogen-exposed groups (HTV and NTV) compared to HTC, and this upregulation was particularly pronounced in the HTV group. *PcSOD3* and *PcSOD5* displayed a distinct temporal pattern, with mid-to-late-stage upregulation peaking at 24 h and 48 h in both HTV and NTV groups compared to HTC, and heat shock pretreatment further augmented their late-phase expression. These results indicate that *SOD* genes in *P. clarkii* exhibit time-specific expression patterns in hemocytes in response to *V. parahaemolyticus* challenge, with heat shock pretreatment modulating the magnitude and timing of these antioxidant-related transcriptional changes (**Figure 6**).

### 3.7 Expression analysis of ERS-related genes in response to bacterial challenge after NLHS

**Figure 7:**
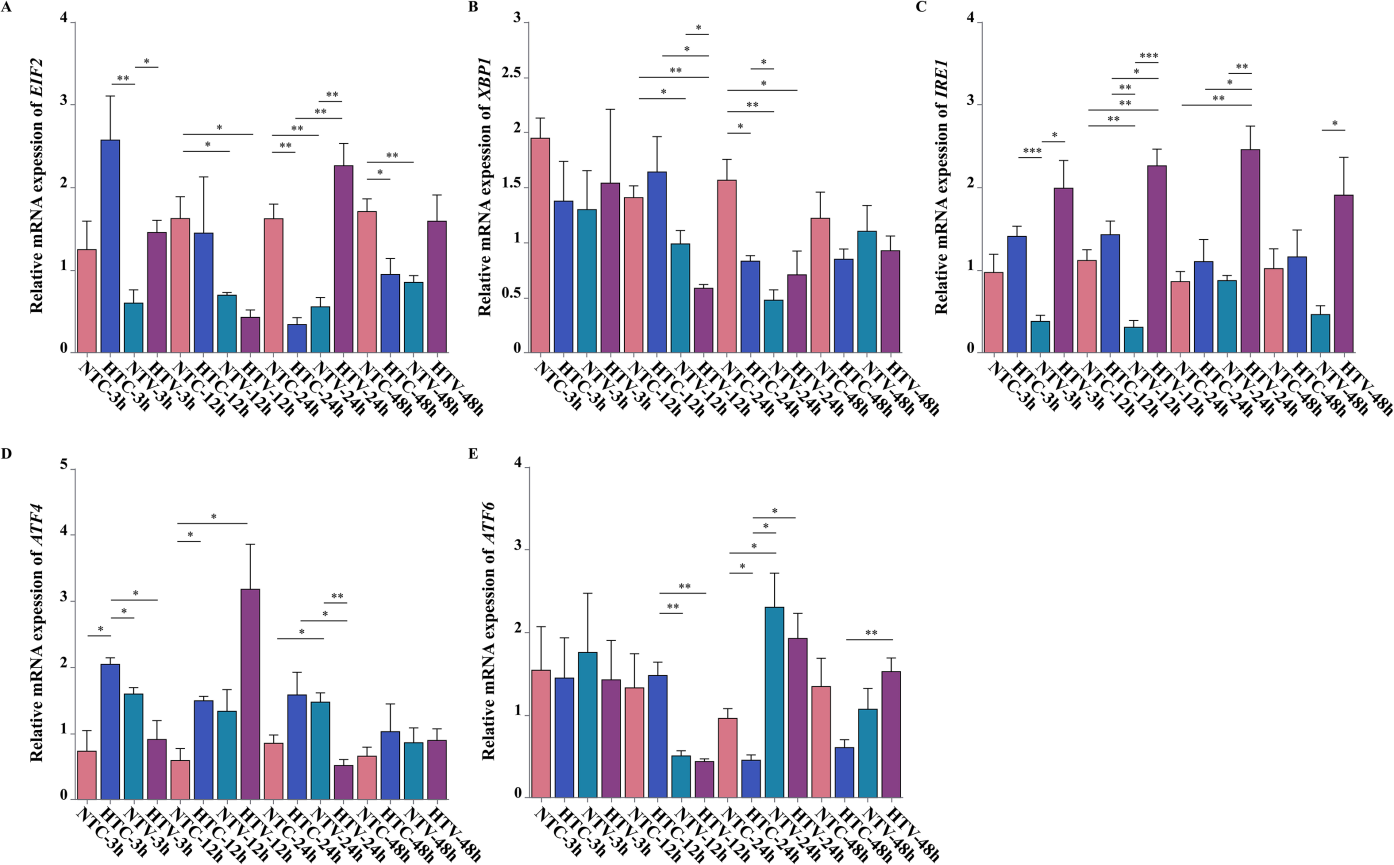
Expression dynamics of ERS-related genes in response to *V. parahaemolyticus* infection following NLHS. **(A–E)** qPCR analysis of *EIF2*, *XBP1*, *IRE1*, *ATF4*, and *ATF6* expression under different treatment groups. Expression levels were normalized to the reference gene *Gapdh*. * represents *p* < 0.05; ** represents *p* < 0.01; *** represents *p* < 0.001.

The expression dynamics of five ERS-related genes (*EIF2*, *XBP1*, *IRE1*, *ATF4*, *ATF6*) in *P. clarkii* hemocytes were analyzed via qPCR after *V. parahaemolyticus* challenge, across different groups and time points. For *EIF2*, pathogen-challenged groups (HTV, NTV) showed significantly higher expression than saline-injected controls (HTC, NTC) at multiple time points, with HTV exhibiting more pronounced upregulation (e.g., 3 h: *p* < 0.01; 12 h and 24 h: sustained difference) (**Figure 7A**). *XBP1* displayed early-to mid-stage upregulation in HTV/NTV versus controls, with HTV showing stronger and prolonged induction (3 h: *p* < 0.05; 12 h and 24 h: *p* < 0.05) (**Figure 7B**). *IRE1* had marked mid-to late-stage upregulation in pathogen-challenged groups, with HTV drastically higher than HTC (12 h: * *p* < 0.001; 24 h and 48 h: *p* < 0.01) (**Figure 7C**). *ATF4* showed a bimodal response: significant upregulation in HTV/NTV versus controls at 3 h (*p* < 0.05, HTV stronger), and a secondary peak at 24 h in HTV (*p* < 0.01) (**Figure 7D**). *ATF6* exhibited late-stage upregulation, with HTV significantly elevated at 24 h (*p* < 0.05) and sustained at 48 h (*p* < 0.01) versus HTC (**Figure 7E**). Collectively, ERS-related genes in *P. clarkii* showed time-specific expression patterns during *V. parahaemolyticus* challenge, with heat shock pretreatment modulating transcriptional response magnitude and timing, reflecting a coordinated ERS defense strategy against pathogenic stress.

### 3.8 Correlation analysis between *SOD* and ERS-related genes in *P. clarkii*

**Figure 8:**
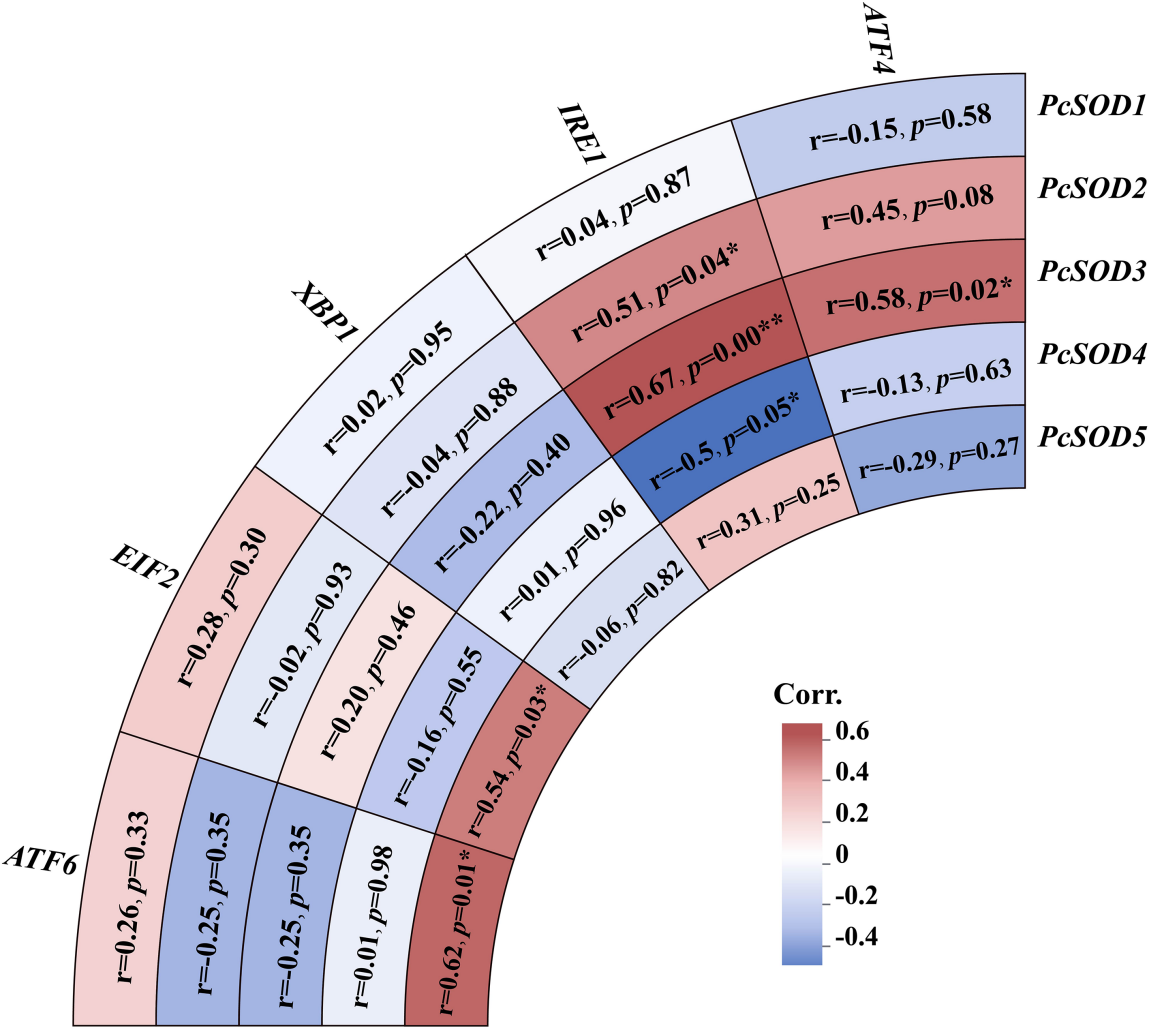
The correlation relationships between *PcSODs* and ERS-related genes predicted to have interaction with them. Color intensity indicates Pearson correlation coefficient (r-value); asterisks denote statistical significance: *p < 0.05, **p < 0.01.

A correlation heatmap was constructed to analyze the expression relationships between five *SOD* genes (*PcSOD1*–*PcSOD5*) and five ERS-related genes (*EIF2*, *XBP1*, *IRE1*, *ATF4*, *ATF6*) in *P. clarkii*. The color scale represented correlation coefficients, with red indicating positive correlations, blue indicating negative correlations, and white/light colors representing no significant correlation. Statistical significance was denoted by * (*p* < 0.05) and ** (*p* < 0.01). Notable positive correlations were observed: *XBP1* showed a highly significant positive correlation with *PcSOD3* (correlation coefficient = 0.00**, *p* < 0.01), suggesting coordinated expression in maintaining cellular homeostasis during stress. *IRE1* was significantly positively correlated with *PcSOD3* (0.04*, *p* < 0.05) and *PcSOD4* (0.05*, *p* < 0.05), implying synergistic responses between the UPR pathway and antioxidant system. Additionally, *ATF6* had a significant positive correlation with *PcSOD3* (0.01*, *p* < 0.05). Some pairs, like *XBP1* with *PcSOD1* (0.88) and *XBP1* with *PcSOD4* (0.96), showed high correlation coefficients without statistical significance, indicating potential associations requiring further validation. These results reveal that *SOD* genes and ERS-related genes do not act independently but form a coordinated defense network, integrating antioxidant functions and ERS responses to cope with pathogenic and environmental stresses in *P. clarkii*. This provides a genetic basis for understanding the interactive regulation of oxidative stress and ERS in crustacean immunity (**Figure 8**).

### 3.9 T-AOC and SOD activity profiles and intergroup differences in *P. clarkii* across treatment groups

**Figure 9:**
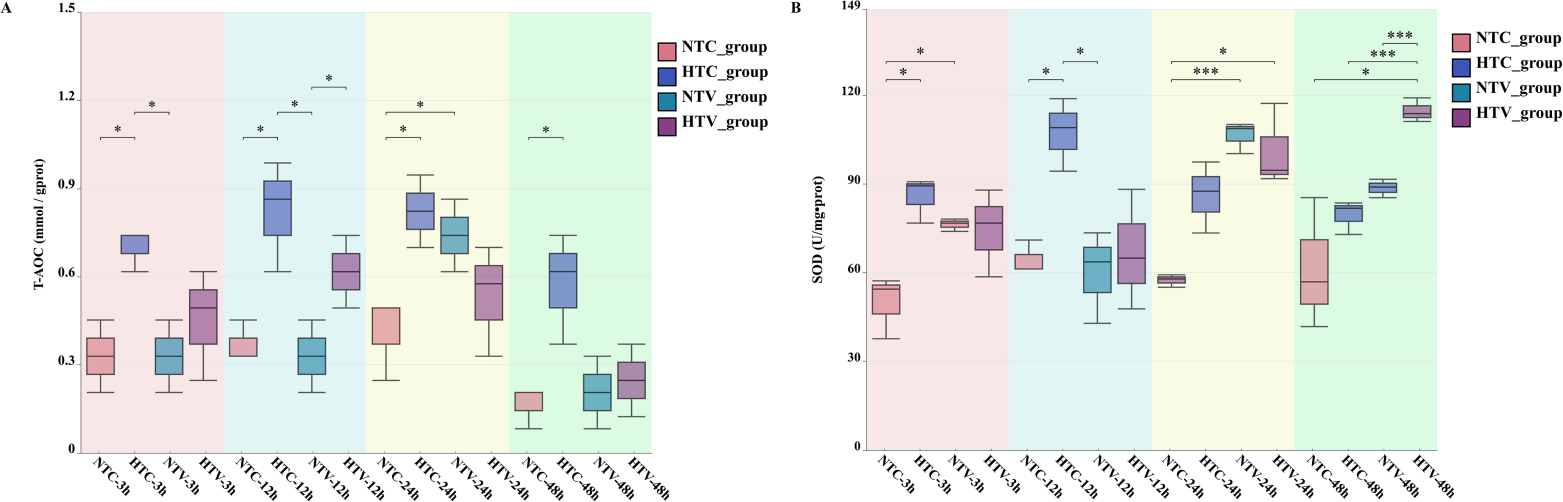
Dynamics of T-AOC and SOD activity in *P. clarkii* in response to *V. parahaemolyticus* challenge following NLHS. **(A)** T-AOC levels and **(B)** SOD activity in hemocytes of *P. clarkii* across four treatment groups (NTC, HTC, NTV, HTV) at 3, 12, 24, and 48 h post-treatment. Statistical analysis was performed using independent samples t-test. Statistical significance is indicated as *p < 0.05 and ***p < 0.001.

T-AOC and SOD activity in *P. clarkii* were measured across four groups (NTC, HTC, NTV, HTV) at 3, 12, 24, and 48 h post-*V. parahaemolyticus* challenge. Pathogen-challenged groups (NTV, HTV) showed significantly higher T-AOC than controls (NTC, HTC) at multiple time points: the HTV group exhibited elevated levels at 3 h (vs NTC, *p* < 0.05; vs NTV, *p* < 0.05) and remained higher at 12 h (vs NTC, *p* < 0.05; vs NTV, *p* < 0.05). Additionally, the HTC group was higher than the NTC group at 12 h (*p* < 0.05), indicating heat shock pretreatment prolonged T-AOC activation. For SOD activity, the HTV group displayed higher activity at 12 h (vs NTC, *p* < 0.05; vs NTV, *p* < 0.05) and sustained elevation at 24 h (vs NTC, *** *p* < 0.001; vs NTV, *p* < 0.05), with the HTC group also higher than the NTC group at 12 h (*p* < 0.05), reflecting that heat shock pretreatment extended SOD-mediated defense. These results indicate that *V. parahaemolyticus* challenge induces time-specific antioxidant activation in *P. clarkii* (increased T-AOC and SOD activity), and heat shock pretreatment enhances the magnitude and duration of these responses, supporting a coordinated defense mechanism against pathogenic stress (**Figure 9**).

## 4 Discussion

As a key freshwater aquaculture crustacean, *P. clarkii* relies heavily on its antioxidant defense system to combat pathogen invasion (34). In this study, we systematically characterized the structural features of the *SOD* gene family in *P. clarkii* at the genomic level, examined their tissue-specific expression profiles, and analyzed their dynamic regulatory responses to bacterial infection. Furthermore, we explored the potential crosstalk between *SOD* family members and ER-stress pathways, highlighting their cooperative roles in immune defense. Notably, we observed that NLHS enhances the antioxidant capacity of *P. clarkii*, and our data suggest that the synergistic regulation between *SOD* genes and ER-stress signaling plays a central role in strengthening resistance against pathogens. These findings offer new insights into the mechanisms of antioxidant-mediated immune regulation in crustaceans.

Regarding the cross-talk between ERS and SOD in *P. clarkii*, our prior work showed NLHS induces Hsp70 upregulation (which enhances *V. parahaemolyticus* resistance via TLR signaling) (29). HSP70 likely acts as a bridge, as it stabilizes ER protein folding to reduce excessive ERS (35). Consistent with crustacean studies (e.g., *L. vannamei*), HSP70 can indirectly regulate antioxidant enzymes, though interspecific regulatory differences may exist (14). Based on the correlation between Hsp70 (29) and PcSOD expression in *P. clarkii*, we infer “ERS may modulate SOD via Hsp70,” with direct causal links to be validated.

In *P. clarkii*, five *SOD* genes whose encoded proteins exhibit considerable variation in amino acid length, molecular weight, and pI were successfully identified. Such differences likely underpin their functional specialization. In *Chlamys farreri*, for example, the Cu/Zn-SOD family has undergone gene expansion, a trait not universal to aquatic invertebrates (36). Our data confirm that *P. clarkii*’s five *SOD* genes have achieved functional specialization via differences in amino acid length and molecular weight. All SOD proteins in *P. clarkii* harbor highly conserved SOD domains, consistent with orthologs in *Pseudosciaena crocea* (37). This structural similarity suggests potential catalytic conservation, though interspecific functional nuances remain unconfirmed.

Phylogenetic analysis classified the five *Pc*SODs into two distinct subfamilies, including Cu/Zn-SODs (*Pc*SOD1–3) and Mn-SODs (*Pc*SOD4–5), consistent with the clustering patterns observed in *Hypophthalmichthys molitrix*, and reflecting evolutionary divergence based on metal cofactors (38). Typically, Cu/Zn-SODs are localized in the cytoplasm or extracellular space, while Mn-SODs are primarily mitochondrial, indicating their distinct roles in oxidative stress responses (39). Homology modeling showed PcSODs feature α-helical and coiled motifs, similar to SODs in *Acipenser baerii* (10).

Notably, chromosomal mapping showed the five *PcSOD* genes are unevenly distributed across four chromosomes, with *PcSOD1* and *PcSOD4* co-localized. This genomic arrangement, not systematically reported in previous studies on *P. clarkii*, may have arisen from tandem duplication events—similar to the chromosomal linkage observed between Cu/Zn-SOD2 and SOD3 in *C. farreri* (36). This co-localization is unique to *P. clarkii*, and compared with the scattered *SOD* gene distribution in *L. vannamei*, the linkage of *PcSOD1* and *PcSOD4* may enable potential synergistic transcriptional regulation under NLHS, facilitating rapid antioxidant responses—this genomic feature may further contribute to *P. clarkii*’s strong environmental adaptability. The co-localization of *PcSOD1* and *PcSOD4* may facilitate coordinated stress-induced expression, consistent with SOD upregulation in *Eriocheir hepuensis*, though stressors and regulatory pathways vary by species (40).

The *PcSOD* genes in *P. clarkii* exhibited distinct tissue-specific expression patterns. *PcSOD4* and *PcSOD5* were highly expressed in hemocytes, *PcSOD1* in the gills, and *PcSOD2*, *PcSOD3*, and *PcSOD4* in the hepatopancreas, reflecting the functional compartmentalization of antioxidant responses in aquatic species. The high expression of *PcSOD4* and *PcSOD5* in hemocytes is consistent with their role in neutralizing ROS generated during immune activation (41, 42). Similar findings have been reported in Pacific abalone, where *HdhCu/Zn-SOD* is upregulated in hemocytes following LPS stimulation (43). In gills, elevated *PcSOD1* expression may protect against waterborne pathogens, similar to the *Mn-SOD* upregulation observed in *H. molitrix* under hypoxia (38). In the hepatopancreas, high levels of *PcSOD2*, *PcSOD3*, and *PcSOD4* likely support its functions in metabolism and detoxification, as also seen in *P. crocea* during *V. alginolyticus* infection (37). This study analyzed seven tissues, expanding on earlier work focused mainly on the hepatopancreas and gills (22). Notably, *PcSOD5* was highly expressed in the intestine, possibly to manage ROS from microbial metabolism, while *PcSOD4* showed low expression in the eyestalk, consistent with lower oxidative stress in neural tissues (22). Low *SOD* expression in the stomach and muscle may relate to oxidative metabolism levels (41), and in muscle, redox balance may instead be maintained by other systems such as glutathione peroxidase (GPx) (44).

After infection with *V. parahaemolyticus* following NLHS, the *SOD* genes in *P. clarkii* displayed a clearly time-dependent expression pattern. During the early response phase (3–12 h), *PcSOD2* and *PcSOD4* were significantly upregulated, particularly in the HTV group, suggesting their involvement in mitigating the acute oxidative burst triggered by pathogen invasion. Components of the pathogen, such as lipopolysaccharides (LPS), are known to activate NADPH oxidase in immune cells, leading to a rapid accumulation of ROS. At this stage, the rapid induction of SOD is essential to prevent oxidative damage (45, 46). A similar response has been reported in *P. crocea*, where Cu/Zn-SOD was upregulated ninefold within 24 h after *V. alginolyticus* infection (37). This parallel does not equate to a conserved mechanism across aquatic animals, instead our qPCR data directly show that *PcSOD2* and *PcSOD4* are specifically upregulated in *P. clarkii* during the early phase 3–12 h of *V. parahaemolyticus* challenge, a species-specific strategy to mitigate acute oxidative bursts. In the middle to late stages of infection (24–48 h), *PcSOD3* and *PcSOD5* remained highly expressed, with the HTV group showing greater upregulation than the NTV group, indicating that heat shock pretreatment enhances sustained antioxidant capacity. As pathogen clearance progresses, physiological processes such as apoptosis and inflammatory cytokine release continue to generate ROS; thus, prolonged *SOD* expression is crucial for maintaining redox homeostasis (47, 48). This temporal pattern mirrors the delayed upregulation of Mn-SOD previously observed in *P. clarkii* following *S. eriocheiris* infection, possibly reflecting the need for mitochondrial repair (22). Notably, *PcSOD1* showed minimal differential regulation across groups and time points, with two potential explanations. On one hand, its predominant expression in gills rather than hemocytes suggests it is not a key subtype for hemocyte-mediated *V. parahaemolyticus* defense. On the other hand, similar to *X. maculatus*, its function may rely on post-translational modifications instead of transcriptional changes, which explains the stable mRNA levels observed (49). These will be verified by future PcSOD1 protein and subtype-specific activity detection.

The regulatory mechanism of NLHS may be associated with HSP cooperation (29). Heat shock induces molecular chaperones like HSP70, which may enhance SOD activity by stabilizing its structure or promoting translation (50). Correlation analysis shows strong positive links between *PcSOD* and ERS genes—*PcSOD3* with *IRE1*/ATF4, and *PcSOD5* with *ATF6*/*EIF2*—suggesting a potential coordinated response. This aligns with *C. carpio* findings (GPx-ERS co-regulation), though causal links need validation (11).

During pathogen-induced oxidative stress, excessive ROS triggers ER unfolded protein response (UPR) (12). UPR factors (XBP1, IRE1) may regulate SOD via shared pathways (e.g., MAPK), forming an “antioxidant–ER protection” response. XBP1 promotes antioxidant gene transcription, while IRE1 attenuates inflammation via TRAF2–JNK; SOD reduces ROS-related cytokines, implying potential synergy (12). Here, XBP1-PcSOD3 correlation suggests possible promoter binding, and IRE1-PcSOD4 may involve the IRE1–TRAF2–JNK axis (12). NLHS-enhanced SOD-ERS correlation may relate to HSP70 stabilizing ER protein folding (51).

T-AOC and SOD activity assays confirm NLHS strengthens the magnitude and duration of SOD responses. This transcriptional upregulation matches enzyme activity increases, supporting NLHS-enhanced pathogen clearance. Notably, this differs from *L. vannamei* (NLHS upregulated HSP70 but not Vibrio resistance), likely due to *P. clarkii*’s unique *PcSOD* dynamics (24). HSP activation may underpin this via SOD folding maintenance and NF-κB activation (52).

In summary, this study comprehensively analyzes the *P. clarkii SOD* gene family, characterizing its key features (gene structure, chromosomal distribution, conserved motifs, tissue-specific expression). Moreover, it provides new insights into the species’ antioxidant defense mechanisms post-NLHS and subsequent *V. parahaemolyticus* challenge. Specifically, it identifies correlative links between NLHS-induced *PcSOD* expression and ERS gene levels in *P. clarkii* hemocytes. These findings provide a valuable genomic resource for crustacean antioxidant research and generate targeted hypotheses to clarify their role in bacterial resistance. This work not only lays a theoretical foundation for understanding how the SOD family enhances *P. clarkii*’s resistance to *V. parahaemolyticus* under NLHS but also reveals potential cooperative mechanisms between antioxidant and stress response pathways. These findings also offer practical implications for pathogen control in *P. clarkii* aquaculture and support sustainable industry development.

## Data Availability

The datasets presented in this study can be found in online repositories. The names of the repository/repositories and accession number(s) can be found in the article/**Supplementary Material**.
