# Supplementary material for: Non-lethal heat shock unlocks SOD gene family diversity for enhanced bacterial resistance in Procambarus clarkii
Source: Front Immunol. 2026 Jan 21;17:1713713. doi: 10.3389/fimmu.2026.1713713 (PMC12867829; doi:10.3389/fimmu.2026.1713713)
Supplement: Supplementary file 1 [file Table1.docx]

Supplementary Material

# Supplementary Tables

**Table S1**

**Primers used in this article**

| **Primer name** | **Nucleotide sequence (5'→3')** | **Purpose** |
| --- | --- | --- |
| *PcSOD1*_F | TCCACGAGTTTGGCGACC | qPCR |
| *PcSOD1*_R | TCACCCACATGGCGGTTC | qPCR |
| *PcSOD2*_F | AGGCTGGAAATCCACCGC | qPCR |
| *PcSOD2*_R | GGGGTTGAAGTGGCCTCC | qPCR |
| *PcSOD3*_F | GCGTTGGTGGCTGGTTTG | qPCR |
| *PcSOD3*_R | GCTCACTTCCGTGCCCTT | qPCR |
| *PcSOD4*_F | ATGGCTCCTGATGCTGGC | qPCR |
| *PcSOD4*_R | CCAACCAGCCCCATCCTG | qPCR |
| *PcSOD5*_F | GGGGAATTGCTGGCAGCT | qPCR |
| *PcSOD5*_R | CCAGCCAACCCCATCCTG | qPCR |
| *XBP1*_F | CCCAAGCATCTGAGCCCC | qPCR |
| *XBP1*_R | TTGCCCTCGTCGTTCACC | qPCR |
| *ATF6*_F | ACGGAACAACTGGGCCAG | qPCR |
| *ATF6*_R | TGGCCCACTGGAGGAGTT | qPCR |
| *ATF4*_F | GCAGGATGGCCAGTCTGG | qPCR |
| *ATF4*_R | TGGCATCGTCACAGCTGG | qPCR |
| *IRE1*_F | GGTGTGGCTGTGGCTCAT | qPCR |
| *IRE1*_R | CAGGGGCGTCGAGAAGAC | qPCR |
| *EIF2*_F | AAGATGTTGCACCCCCGG | qPCR |
| *EIF2*_R | CCACGGAGGACCAAGCAG | qPCR |
| *Gapdh_*F | GCCCAGAACATCATCCCATCT | qPCR |
| *Gapdh*_R | CGTCATCCTCAGTGTAACCCAAG | qPCR |
